# Improving Diagnostic Accuracy in Respiratory Allergy: Monocentric Reevaluation of the GA^2^LEN Panel in Germany

**DOI:** 10.1002/clt2.70102

**Published:** 2025-09-24

**Authors:** Caroline Beutner, Christian Meyer, Moritz Maximilian Hollstein, Katharina Klara Hahn, Michael Peter Schön, Timo Buhl

**Affiliations:** ^1^ Department of Dermatology Venereology and Allergology University Medical Center Göttingen Göttingen Germany; ^2^ Software Development on behalf of Information Network of Departments of Dermatology (IVDK) University of Göttingen Göttingen Germany

**Keywords:** aeroallergens, sensitization, skin prick test panel

## Abstract

**Background:**

European guidelines recommend using a standardized baseline series of skin prick test (SPT) allergens for the diagnosis of airway allergies. In addition, local adaptation and/or extension of test panels according to regional exposure and sensitization patterns are increasingly being discussed.

**Methods:**

Regional sensitization rates according to SPTs based on the most recent GA^2^LEN baseline series in Central Germany were retrospectively analyzed for 960 consecutive patients with respiratory symptoms at our university outpatient clinic. Additional SPT allergens of interest were further analyzed.

**Results:**

High sensitization rates to the baseline SPT series were observed in our highly selected study cohort. The positivity rates were particularly high for olive pollen (30.8%) and plantain pollen (33.4%). Positive olive and birch pollen SPTs were found in 98.5% of olive‐ash‐birch pollen tested patients. High SPT positivity rates (98.1%) for plane tree and olive tree pollen were found, whereas only six patients (1.9%) were diagnosed with exclusive cypress pollen sensitization. Subgroup analysis of SPTs for palm tree pollen revealed that 92% of patients with palm tree positivity showed polysensitization, and all but one patient had concomitant grass pollen sensitization.

**Conclusion:**

Regular evaluations of SPT series may be necessary because of climate change, extract production, and increasing population mobility. Ash and cypress pollen extracts could currently be removed from the baseline SPT panel without significantly decreasing diagnostic accuracy. Positive SPTs to non‐native palm tree pollen may indicate the presence of IgE to cross‐reacting panallergens, which may help to differentiate primary sensitization from cross‐reactivity directly. Limitations include the retrospective monocentric design and lack of molecular IgE confirmation.

**Abbreviations**
GA^2^LEN: Global Allergy and Asthma European Network

## 1 Introduction

Representative population‐based national sensitization rates to aeroallergens in Germany range from 34% in adults (*German Health Interview and Examination Survey for Adults* DEGS1 study, 2008–2011) to 36% in children and adolescents (German Health Interview and Examination Survey for Children and Adolescents [KIGGS] baseline study, 2006–2009), according to serological screening for frequent aeroallergens [1]. The skin prick test (SPT) is an inexpensive and simple method for diagnosing IgE‐mediated respiratory diseases. On the basis of a comparison of European SPT procedures and sensitization rates [2], the compilation of a minimum battery of aeroallergens has been recommended to standardize SPTs [3]. Furthermore, pragmatic limitation of the aeroallergen baseline panel to the required allergens is cost‐effective, and facilitates testing implementation when test allergen availability and market approval are limiting factors [4, 5]. One challenge in compiling a Europe‐wide SPT baseline series is local changes in aeroallergen sensitization, because of geographic and climatic differences [6, 7]. Simultaneously, climate change, altered pollen counts, and increasing population mobility may lead to novel sensitization patterns. Lack of inclusion of rare and regionally underestimated allergens can therefore lead to diagnostic gaps [3, 8, 9, 10, 11]. Minimal screening aeroallergen batteries in children and adults with allergic airway diseases have been investigated in international studies considering local sensitization rates [12, 13]. The replacement of allergens by representatives of homolog groups [14], and the benefit of testing multiple allergens of the *Oleacea* family (as recommended for Europe) [3] have sparked novel discussions in Germany [9, 15]. The inclusion of ash pollen extract in the baseline SPT series has also been debated for Germany, and this allergen is currently not part of the Global Allergy and Asthma European Network (GA^2^LEN) baseline series recommendation. Observations of increasing olive pollen sensitization are likely to have resulted from cross‐reactivity to ash pollen [16, 17]. Olive tree pollen, the major representative of the *Oleaceae* family, is predominantly responsible for airway allergies in the Mediterranean [18]. Approximately 15 years ago, the GA^2^LEN skin test study III led to the continued recommendation of testing for 18 allergens in clinical practice in Europe. Further panel reductions generally led to the identification of patients with aeroallergen sensitivity, but important details regarding patient‐specific sensitization patterns were lacking. Understanding individual and clinically relevant sensitization patterns including cross‐reactivity is essential for optimizing personalized therapy decisions [19]. In the Europe‐wide GA^2^LEN SPT study from 2009, the participation of only one or two study centers per country (including centers in Munich and Berlin for Germany) limited the conclusions. Other limitations included possible cross‐reactivity without differentiation and the lack of assessment of clinical relevance. Given the evolving aeroallergen landscape due to climate variability and migration, and the more than 15‐year gap since the last GA^2^LEN recommendations, a regional update of diagnostic panels is both timely and necessary.

Most patients with relevant airway allergies are known to have polysensitization [20, 21]. However, polysensitization in aeroallergen SPT is often attributed to cross‐reactivity to pan‐allergens, primarily those of plant origin [22]. Detection can be performed through serological investigation of molecular allergens, which is rarely performed outside specialized centers, because of time and cost constraints. An interesting candidate for detection of pan‐allergen sensitization directly in SPTs may be a plant extract that does not show clinically relevant sensitization rates in the geographical region. Palm trees, belonging to the monocot family, contain profilins as their major allergens and elicit clinically relevant sensitization rates of 19%–25% in southern European regions. This extract is currently under discussion as a potential profilin marker for patients with pollen polysensitization in Central Europe [23].

Herein, we investigated recent sensitization rates in a large study cohort in Central Germany, according to findings from SPTs based on the recommended baseline series from the 2009 GA^2^LEN study III. We also investigated the local relevance of the rare pollen allergens of plane trees, olive trees, and cypress trees, to propose changes to the number of test allergens and the composition of the baseline SPT series in Germany. The sensitization patterns of patients with palm tree pollen positivity were separately evaluated, to evaluate this pollen extract as a potential pan‐allergen indicator for Central Europe.

## 2 Materials and Methods

SPTs were retrospectively analyzed in 1020 consecutive patients presented with respiratory symptoms suggestive of allergic rhinitis and/or asthma at the Department of Dermatology, Venereology and Allergology at the University Medical Centre Göttingen (UMG) in Germany between 2017 and 2022. Göttingen, a city of 135,000 inhabitants and a catchment area of approximately 1.5 million people, is located close to the geographical center of Germany. This study was conducted in accordance with the Declaration of Helsinki and was approved by the institutional review board of the ethics committee of the UMG. The baseline SPT panel included birch pollen, alder pollen, hazel pollen, grass pollen mix, *Dermatophagoides pteronyssinus* (*D.p.*), *Dermatophagoides farina* (*D.f.*), cat, dog, ash pollen, olive pollen, plane tree pollen, cypress pollen, plantain pollen, *Artemisia*, *Ambrosia*, *Parietaria*, *Alternaria*, *Aspergillus fumigatus*, and *Cladosporium herbarum*. We did not include *Blatella*, because of lack of clinical relevance, and we added plantain pollen, because of reports of increasing relevant sensitization in Germany, in accordance with the recommendation to adapt the individual allergens in the standardized series according to regional conditions [7, 24, 25]. SPTs were performed according to current guidelines by only four experienced nurses, thus reducing inter‐individual bias due to differences among evaluators. SPT was performed as a baseline series according to the guidelines of the German Society for Allergology and Clinical Immunology on the volar forearm, wheal diameter cutoff (≥ 3 mm) was rated as positive results, suppressive medications with respect to the SPT were paused with recommended interval (DGAKI) [26]. Histamine dihydrochloride (Allergopharma, Reinbek, Germany) served as a positive control, and the diluent (Allergopharma, Reinbek, Germany) served as a negative control. All standardized commercial pollen extracts of the SPT series except for cypress pollen (Leti Pharma, Witten, Germany) were obtained from Allergopharma (Reinbek, Germany). A total of 960 patients were included (Table 1), whereas patients with negative histamine or positive NaCl control or repetitive testing were excluded (*n* = 60 patients).

**TABLE 1:** Age‐ and sex distribution of the study group stratified by suspected type I hypersensitivity (airway allergy), with and without coexisting type IV hypersensitivity (e.g., contact dermatitis).

Patient age (years)	Total number	Male (*N*)	Female (*N*)	Type I hypersensitivity suspected (*N*)	Type I + IV hypersensitivity suspected (*N*)
00–09	7	3	4	6	1
10–19	106	49	57	103	3
20–29	167	56	111	161	6
30–39	162	58	104	149	13
40–49	172	57	115	162	10
50–59	187	66	121	164	23
60–69	106	34	72	97	9
70–79	46	19	27	37	9
80–89	7	4	3	7	0

**FIGURE 1:**
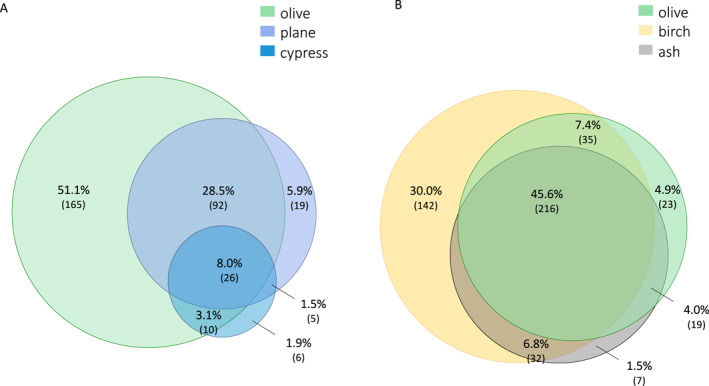
Mono‐ and cross‐sensitization in two subgroup analyses of the study cohort. (A) Sensitization patterns are depicted to olive, cypress, and plane tree pollen with at least one allergen with a positive test result in a Venn diagram to show overlapping positive reactions (*n* = 323 patients). (B) Sensitization patterns to birch, ash, and olive pollen are shown with at least one allergen with a positive test result in a Venn diagram (*n* = 474 patients).

Aeroallergen sensitization rates were evaluated, and cross‐sensitization patterns of special interest were analyzed in detail (pollen from cypress, olive, plane as well as pollen from birch, ash, olive). Cross‐sensitivity is depicted in Venn diagrams showing patients with at least one positive test for these three allergens. Finally, we performed subgroup analyses of SPTs with palm tree pollen as a possible polysensitization marker for plant panallergens (which we have included in our test series since mid‐2021). The main plant allergen groups were a grass pollen group (tested with a grass pollen mixture), tree pollen group (tested with birch, alder, and hazel pollen), and weed pollen group (tested with *Artemisia*, *Ambrosia*, *Parietaria*, and plantain). Palm tree pollen was obtained from Leti Pharma (Leti Pharma, Witten, Germany). For patients with palm tree pollen positivity, co‐sensitization patterns to the allergens of the three plant groups were separately analyzed in detail. This primarily descriptive study included statistical analyses using R 4.2.1 (R Project; www.r‐project.org). For group comparisons in Figure 1, we applied fisher tests using the using R stats package 4.2.1 and the fisher test function.

## 3 Results

### 3.1 Sensitization Rates in the Baseline Series for Diagnosis of Airway Allergies

**FIGURE 2:**
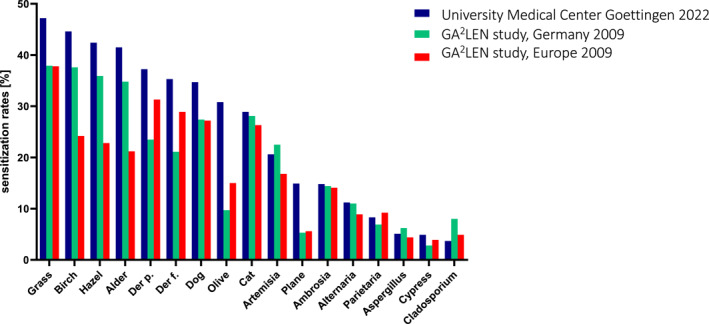
Positive SPT results to allergens in the baseline series, at present and in the GA^2^LEN study in 2009. Positivity rates of baseline series SPTs in symptomatic patients at our study site (University Medical Center Goettingen, UMG) in Germany (blue) are shown. Prior results from the GA^2^LEN studies in 2009 are reproduced for Germany (green) and all European centers (red) [16].

The total study cohort comprised 960 patients (Table 1): 614 women (41.9 ± 17.0 years) and 346 men (41.1 ± 18.2 years). In our cohort, the highest sensitization was found for grass pollen (47.2%) and tree pollen (birch: 44.6%; hazel: 42.4%; and alder: 41.5%) (Figure 2). Figure 2 compares our data with the SPT results from the large patient‐based GA^2^LEN study published in 2009. Our current study cohort showed greater sensitization to *D.p.* than *D.f.*, and lower sensitization rates to cat than dog. We observed a high sensitization rate of 30.8% for olive tree pollen, which exceeded that for the botanically related ash tree (28.7%; data not shown in Figure 2, because ash pollen was not investigated in the GA^2^LEN trials). We found that 14.9% of patients had positive SPTs for plane tree pollen, and 4.9% of patients reacted to cypress pollen. In the weed group, plantain (data not shown) showed the highest sensitization rate (33.4%) and was followed by *Artemisia* (20.6%), *Ambrosia* (14.8%), and *Parietaria* (8.3%) (Figure 2).

### 3.2 Sensitization Patterns to Pollen Allergens of Special Interest

Next, we focused on SPT patterns for three allergen extracts—olive, plane tree, and cypress pollen—because pollen from these plants may show some cross‐reactivity. Only patients with at least one positive SPT result to these three allergens (*n* = 323) were selected. A total of 239 patients (90.7%) showed sensitization to olive pollen, most of whom (51.1%) showed mono‐sensitization to olive pollen among these three allergens (Figure 1A). Monosensitization to these three allergens was significantly more common than all double sensitizations (olive pollen compared to co‐sensitization to plane or zypress pollen; *p* < 0.001 each), as well as triple sensitizations (olive pollen with co‐sensitization with cypress and plane; *p* < 0.001). Most patients with plane tree pollen sensitization simultaneously showed sensitization to olive pollen (significantly more frequent compared to co‐sensitization with plane and olive pollen; *p* < 0.001). Among these patients, 28.3% additionally showed sensitization to cypress pollen. Only several patients showed mono‐sensitization to plane tree pollen, and even fewer (*n* = 6) patients showed sensitization exclusively to cypress pollen. Patients with positive SPTs to cypress pollen frequently showed cross‐reactivity to plane tree and olive pollen without significant difference to double‐sensitization patterns containing cypress pollen (Figure 1A). In a second analysis, we examined the subgroup of patients with positivity for at least one SPT for ash, birch, and olive pollen (*n* = 474). As expected, most of these patients were sensitized to birch pollen (Figure 1B). We observed a remarkably high poly‐sensitization rate to all three allergens tested (45.6% of all patients in the substudy). Mono‐sensitization was significantly higher to birch pollen (30.0%) compared to rarely found mono‐sensitization to olive pollen (4.9%; *p* < 0.001) as well as ash pollen (1.5%; *p* < 0.001).

### 3.3 Improving the Diagnostic Accuracy of SPT by Including a Cross‐Reactive Allergen Such as Palm Tree Pollen

**FIGURE 3:**
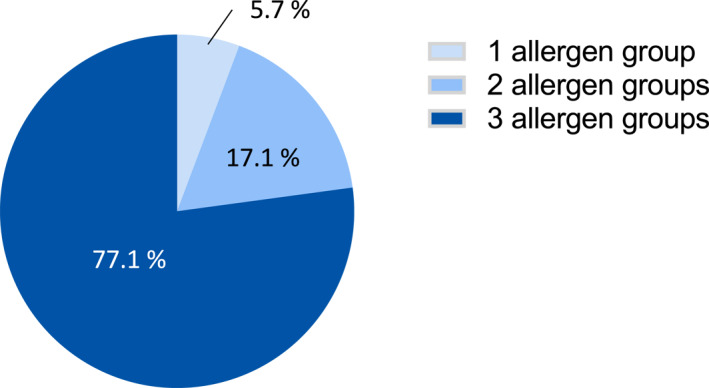
Patients with palm tree positivity and additional sensitization to the plant‐based aeroallergen groups. Sensitization patterns to trees, grass, and/or weeds with at least one allergen with a positive test result in addition to a positive SPT to palm tree (*n* = 35 patients). Tree pollen (positive SPT to at least one of birch, alder, hazel, and ash pollen), grass (positive SPT to grass mix), and weeds (positive SPT to at least one of *Artemisia*, *Ambrosia*, *Parietaria*, and plantain) were investigated.

**FIGURE 4:**
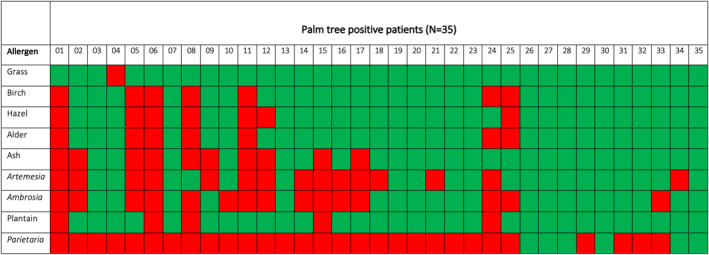
Detailed co‐sensitization patterns of patients with palm tree pollen SPT positivity. Green boxes represent positive SPTs, and red boxes represent negative test results in 35 patients with palm tree positivity. Patients were numbered consecutively (no. 01–35).

Profilin‐rich palm pollen has been discussed as an indicator for pollen panallergens in regions where palm trees are not native. We observed SPT positivity for palm tree pollen in 16.3% of tested patients. Analysis of poly‐sensitization patterns revealed that 94.2% (*n* = 33/35) of patients with palm pollen positivity showed additional sensitization to at least two major allergen groups among the three plant‐originating allergen groups (tree, grass, and weed pollen; Figure 3). In individual patients, high cross‐reactivity was observed between closely related allergens (Figure 4). In 77.1% (*n* = 27/35) of patients with palm tree positivity, we detected poly‐sensitization to the three plant‐originating allergen groups, whereas only two individuals were mono‐sensitized to grass pollen (Figure 4). Examination of the individual patients' results from the palm positive subgroup revealed that all but one patient showed sensitization to grass pollen, 82.9% showed sensitization to plantain pollen, and 77.2% showed sensitization to tree pollen (birch, alder, and/or hazel pollen). The lowest co‐sensitization rate among patients with palm pollen positivity was found for *Parietaria* (17.0%; Figure 4).

## 4 Discussion

Our findings raise important questions about the diagnostic value and clinical utility of current SPT panel compositions, particularly in Central Europe. Highest sensitization rates to grass and tree pollen in our study cohort were in line with previously described data from Central Germany in 1998–2017 [27] and from the two German study centers participating in the GA^2^LEN studies in 2009 (Figure 2). The high sensitization rate of 30.8% for olive tree pollen, which was more than twice that in the GA^2^LEN study, might be explained by cross‐reactivity, given the high birch pollen sensitization rate of 44.6% in our study cohort. Equally high SPT rates in 2001–2013 for olive pollen have been reported for South Germany (41%) together with high birch pollen sensitization (> 65%) [8]. Interestingly, high birch pollen sensitization (37.6%) in the German subgroup of GA^2^LEN patients was not associated with a high olive pollen sensitization rate (9.7%) at that time. One possible explanation for the differing findings might be differences in the olive pollen extracts among various manufacturers [28]. The fairly high ash pollen sensitization rate of 28.7% in our cohort (data not shown) is within the range reported for South and West Germany more than 10 years ago (22%–33%) [8]. The same authors reported slightly lower positivity rates in Germany for plane tree pollen (currently 14.9% in our study) more than 10 years ago. Of note, the SPT findings for plane tree pollen from 2009 were much lower than the current values, probably due to crossreactivity from high grass pollen sensitization via profilins in our cohort, and as previously hypothesized [23].

Analysis of the first special interest SPT patterns for olive, ash, and birch pollen‐tested patients indicated that 18.6% showed olive pollen sensitization but not ash pollen sensitization, in agreement with German data indicating rates of 8%–21% more than a decade ago. Known high cross‐reactivity to ash and olive pollen was also confirmed in our study cohort (24.7% of all patients tested), in agreement with earlier publications [8]. Although ash pollen is endemic, primary birch and olive pollen sensitization were likely responsible for the positive SPT to ash pollen observed in our study cohort. One explanation for our higher sensitization rates to the olive tree pollen family compared with older data might have been due to our use of optimized SPT extracts. Changes in pollen exposure due to increasing global mobility and climate change, thereby leading to different and longer allergenicity periods, might also explain the increased rates [29]. Patients with ash‐independent olive pollen positivity, as described several years ago, face a diagnostic gap when the latest GA^2^LEN baseline series from 2009 is used. We agree with earlier publications suggesting that allergens of the *Oleaceae* family should be included [8].

The sensitization patterns of the second special interest topic of cypress, olive, and plane tree pollen‐tested patients demonstrated high olive pollen sensitization, whereas only 9.3% of the patients showed mono‐sensitization or double sensitization (cypress/plane tree pollen without olive pollen sensitization). Among all sensitized patients in this subgroup, 98.1% showed olive and plane tree pollen sensitization. The previously reported 8% rate of serological sensitization to cypress pollen was explained primarily by cross‐reactivity. Another large German SPT study has revealed even fewer positive results to cypress pollen (3/952 patients) [30]. Our analysis of weeds, *Artemisia*, *Ambrosia*, and *Parietaria* indicated almost identical total rates in our study cohort and the 2009 and 2011–12 studies from Germany [8]. Plantain, which is not recommended in the Pan‐European Ga^2^LEN panel, notably showed the highest rate of 33.4% positive SPT results, in accordance with our previously published findings [25]. Furthermore, our data indicated that ash and cypress pollen may be possibly adjustable in the core baseline SPT series for airway allergies in Germany, but should remain available for regionally adapted modules and extended SPT series. These tests may be useful in exceptional cases. Because of high local plantain sensitization rates, replacement of the weed *Parietaria* (8.3%), for which tests are rarely positive, should be considered. We previously published data on potentially decreasing parallel testing of homologous allergens in the German SPT baseline series [31].

Sensitization to the panallergen groups of profilins and polcalcins may complicate SPT interpretation, particularly in patients with positive SPT results for the three main plant‐based aeroallergen groups. Currently, the non‐native palm tree has been discussed as a suitable panallergen marker in Central Europe, because of its high profilin content [32]. Although palm tree pollen reactivity in SPT has been described for individual cases in Germany [23], larger studies are lacking. Herein, 16% of our patients showed palm tree pollen sensitization, 92% of whom were sensitized to at least two larger plant‐based aeroallergen groups. Sensitization to profilin/polcalcin in Central Europe is due primarily to grass sensitization, in line with our data indicating that all but one patient sensitized to grass pollen showed additional palm tree pollen SPT positivity. Additional benefits may be gained by using palm tree pollen SPTs, given that this tree is considered non‐native in Germany. This extract, containing primarily profilin, appeared to be an appropriate panallergen indicator in our study cohort. We recommend that positive palm tree SPTs should trigger serological determination of molecular allergens, because multiple positive SPTs to plant‐based aeroallergens might not indicate primary sensitization. It is important to note SPT positivity does not equate to clinical allergy and no symptom scores or correlation with clinical outcomes were available, which limits conclusions regarding diagnostic performance. Therefore, confirmatory provocation testing would be required to assess clinical relevance. Nevertheless, including an allergen such as palm tree pollen in SPTs to identify patients with panallergen sensitization would immediately increase diagnostic accuracy for patients with airway allergies to identify cross‐sensitivity, at low cost, because such patients are rarely serologically diagnosed in real‐life diagnostic procedures. However, we cannot exclude rare cases of true palm tree co‐sensitization in our study cohort, because of the lack of availability of serological parameters. The findings of our study are of exploratory nature and molecular diagnostics including pollen major allergens should be considered in further studies to distinguish complex pattern of sensitization and genuine sensitization in correlation of clinical relevance. While palm tree pollen may indicate profilin sensitization, its diagnostic role remains speculative without molecular IgE confirmation and requires prospective validation.

## 5 Conclusion

Our data support at least a regionally adapted revision of the GA^2^LEN baseline SPT panel from 2009, particularly regarding ash, cypress, and plantain pollen. Updates of the 2009 European version of the GA^2^LEN baseline SPT panel may be considered. Limitations of this study include the lack of year‐wise sensitization trends and serological (molecular) data, the retrospective study design, a monocentric setting and the predominantly descriptive nature of the analysis. Focusing on Clinical and Public Health Implications, our findings may influence future diagnostic and therapeutic approaches resulting in regional SPT panel updates in Germany and consecutive implications of tailored allergen immunotherapy formulation. Future prospective studies with molecular diagnostics are needed to validate palm tree pollen as a panallergen marker and refine diagnostic strategies in respiratory allergy.

## Author Contributions

**Caroline Beutner:** conceptualization, investigation, writing – original draft, methodology, writing – review and editing, data curation, supervision, project administration. **Christian Meyer:** writing – review and editing, software, formal analysis. **Moritz Maximilian Hollstein:** writing – review and editing. **Katharina Klara Hahn:** writing – review and editing. **Michael Peter Schon:** writing – review and editing. **Timo Buhl:** conceptualization, investigation, writing – original draft, methodology, writing – review and editing, project administration, data curation, supervision.

## Ethics Statement

The study was approved by the ethics committee of the University Medical Center Göttingen, under reference number 2/10/2022. The research was conducted in accordance with the World Medical Association's Declaration of Helsinki.

## Consent

All authors reviewed the final manuscript version and consented to its submission.

## Conflicts of Interest

The authors declare no conflicts of interest.

## Data Availability

All data generated or analyzed during this study are included in this article. Further inquiries may be directed to the corresponding author.
